# Complete Genome Assembly of *Streptococcus pyogenes* ATCC 19615, a Group A β-Hemolytic Reference Strain

**DOI:** 10.1128/genomeA.00976-14

**Published:** 2014-09-25

**Authors:** T. D. Minogue, H. A. Daligault, K. W. Davenport, K. A. Bishop-Lilly, S. M. Broomall, D. C. Bruce, P. S. Chain, O. Chertkov, S. R. Coyne, T. Freitas, K. G. Frey, H. S. Gibbons, J. Jaissle, C. L. Redden, C. N. Rosenzweig, Y. Xu, S. L. Johnson

**Affiliations:** a Diagnostic Systems Division (DSD), United States Army Medical Research Institute of Infectious Diseases (USAMRIID), Fort Detrick, Maryland, USA; b Los Alamos National Laboratory, Los Alamos, New Mexico, USA; c Naval Medical Research Center (NMRC)-Frederick, Fort Detrick, Maryland, USA; d Henry M. Jackson Foundation, Bethesda, Maryland, USA; e United States Army Edgewood Chemical Biological Center (ECBC), Aberdeen Proving Ground, Aberdeen, Maryland, USA

## Abstract

We present the complete genome assembly of *Streptococcus pyogenes* ATCC 19615 (Rosenbach) as submitted to GenBank under accession number CP008926. This group A nonmotile β-hemolytic clinical isolate is used for quality control in a variety of commercially available tests. The assembled genome is 1.84 Mb (38.5% G+C content) and contains 1,788 coding regions.

## GENOME ANNOUNCEMENT

*Streptococcus pyogenes* infections are highly diverse symptomatically and include soft tissue infections, toxic shock syndrome, and pneumonia (1). Notably, this organism is the cause of up to 30% of childhood acute bacterial pharyngitis cases (2), and up to 20% of asymptomatic children may be carriers at various times of the year (3).

High-quality genomic DNA was extracted from a purified isolate using a Qiagen Genomic-tip 500 at the USAMRIID Diagnostic Systems Divisions (DSD). Specifically, a 100-mL bacterial culture was grown to stationary phase and nucleic acid extracted as per manufacturer’s recommendations. Sequence data generated include both Illumina (standard unpaired 100-bp library at 1,000-fold genome coverage) and Roche 454 (7,892- ± 1,973-bp insert, 62-fold genome coverage) data types (4, 5). The two libraries were assembled together in Newbler (Roche) and the consensus sequences computationally shredded into 2-kbp overlapping fake reads (shreds). The raw reads were also assembled in Velvet, and those consensus sequences were computationally shredded into 1.5-kbp overlapping shreds (6). Draft data from all platforms were then assembled together with Allpaths and the consensus sequences computationally shredded into 10-kbp overlapping shreds (7). We then integrated the Newbler consensus shreds, Velvet consensus shreds, Allpaths consensus shreds, and a subset of the long-insert read pairs using parallel Phrap (High Performance Software, LLC). Possible misassemblies were corrected and some gap closure accomplished with manual editing in Consed (8–10).

Automatic annotation for the *S. pyogenes* ATCC 19615 genome utilized an Ergatis-based workflow at LANL with minor manual curation. The complete annotated genome assembly is available in NCBI and raw data can be provided upon request. This finished assembly includes one chromosome (1,844,804-bp, 38.5% G+C content) that contains 1,788 CDS, 18 rRNAs, and 67 tRNAs. Two M proteins are encoded, serotypes 5 and 49.

### Nucleotide sequence accession number.

The annotated genome assembly of *S. pyogenes* ATCC 19615 is available in GenBank under accession number CP008926.
